# Genomic analysis of a novel ST11(PR34365) *Clostridioides difficile* strain isolated from the human fecal of a CDI patient in Guizhou, China

**DOI:** 10.1515/biol-2025-1067

**Published:** 2025-05-20

**Authors:** Ying Yang, Luhong Shu, Ping Ling, Junyi Yang, Ruirui Shao, Yumei Cheng, Shanshan Luo, Xinglang Wei, Zhizhong Guan, Zhenghong Chen, Jian Liao, Xiaolan Qi, Guzhen Cui, Wei Hong

**Affiliations:** Key Laboratory of Endemic and Ethnic Diseases, Ministry of Education & School/Hospital of Stomatology & Key Laboratory of Microbiology and Parasitology of Education Department of Guizhou, Guizhou Medical University, Guiyang, Guizhou, 550025, China; Pediatric Intensive Care Unit & Guiyang Maternal and Child Health Care Hospital, Guizhou, 550003, China; Department of Critical Care Medicine, The Affiliate Hospital of Guizhou Medical University, Guiyang, 550004, China; Collaborative Innovation Center for Prevention and Control of Endemic and Ethnic Regional Diseases Co-constructed by the Province and Ministry, Guiyang, Guizhou, 550025, China; School/Hospital of Stomatology, Guizhou Medical University, Guiyang, Guizhou, 550025, China

**Keywords:** *Clostridioides difficile*, *C. difficile* infection, genomic sequencing, gene annotation, phylogenetic tree, comparative genomic analysis

## Abstract

*Clostridioides difficile* is a pathogen that causes pseudomembranous colitis with antibiotic-associated diarrhea. The epidemiology and molecular evolution of *C. difficile* may differ among different geographic regions, and mining its genomic information can help to understand the epidemiology and molecular evolution of *C. difficile* and focus on its transmission mode. A *C. difficile* strain denoted WXL8 was isolated from a human fecal sample from a patient in the intensive care unit, and its physiology and genomic sequence were determined. The total genome size of WXL8 was 4,119,929 bp, and the GC content was 27.97%. The multilocus sequence typing results indicated that WXL8 is strain type 11 (ST11), a genotype widely present in livestock. The WXL8 was located in clade 5 of ST11. The ribotype of WXL8 was a novel ribotype (PR34365). It is the first report of the ST11 (PR34365) strain. Comparative genomic analysis between WXL8 and the other four high-virulence strains (CD630, CDBR81, CDS-0253, and CDR20291) showed differences in gene arrangement, indicating the uniqueness of *C. difficile* WXL8. In the present study, a novel ribotype (PR34365) ST11 strain of *C. difficile* was isolated from a patient with diarrhea in Guizhou, China. Our findings suggest that zoonotic CDI should receive more clinical attention.

## 1 Introduction

*Clostridioides difficile* is an obligate anaerobic, Gram-positive, spore-forming bacterium found ubiquitously in the environment and the gastrointestinal tracts of humans and animals. It is primarily transmitted through the fecal–oral route [1,2]. *C. difficile* is the primary pathogen causing antibiotic-associated diarrhea (AAD) in the hospital environment, and it is closely related to the disorder of gut microbiota caused by the use of antibiotics [3,4]. A recent report from the Center for Disease Control and Prevention ranks *C. difficile* infection (CDI) as the urgent public health threat with 223,900 incidences and 12,800 deaths yearly and the cost of bed days and associated treatment totaling $1 billion. Recently, *C. difficile* has exceeded multi-drug resistant organisms such as methicillin-resistant *Staphylococcus aureus* as the urgent-control needed pathogen causing hospital-acquired infection [5,6]. Moreover, the recurrent rate of CDI is estimated to occur at 20–30% [7], which together imposes an extreme burden on the healthcare system worldwide.

A critical development in *C. difficile*’s molecular evolution is the emergence of binary toxin-producing ribotypes, such as 027 and 176, associated with more severe clinical presentations and higher recurrence rates than non-binary producers. The rapid dissemination and clonal spread of hypervirulent strains like NAP1/RT027 have amplified concerns regarding CDI’s global prevalence, with these strains spreading across Europe, North America, Asia, and Australia [20]. In recent years, there have been substantial changes in *C. difficile*’s global epidemiology and molecular characteristics. Predominant ribotypes such as 027 and 126 have been replaced by new, highly pathogenic strains with increased virulence factors, resistance profiles, and transmission rates. Ribotype 078 has emerged as a significant cause of CDI outbreaks in several countries, emphasizing the need for continuous monitoring and surveillance [21,22]. The genetic plasticity within *C. difficile*’s pan-genome has facilitated the exchange of virulence factors and antimicrobial resistance genes among strains, aiding their adaptation in various environments [23]. Transferable *C. difficile* plasmids harboring antibiotic resistance determinants have been identified in both human and animal populations, highlighting the zoonotic potential of this bacterium. The expanding knowledge on global *C. difficile* epidemiology and its molecular evolution provides a crucial context for understanding the complex interplay between this bacterium, its human and animal hosts, and the environment [24]. Positioning studies within this broader framework can offer valuable insights into *C. difficile*’s emergence, transmission, and virulence factors, contributing to improved prevention and control strategies in public health and veterinary medicine.

Genotyping methods are a valuable tool for studying the molecular epidemiology of *C. difficile*. These methods can help to clarify the genotypes of highly virulent *C. difficile* strains that are prevalent worldwide [8]. *C. difficile* sequence type 11 (ST11) is one of the most prevalent and pathogenic sub-lineages of *C. difficile* [9]. It is primarily associated with livestock (such as chickens, ducks, and pigs) and food contamination [10,11]. In a study of 953 animal fecal samples from China, Zhang et al. isolated 55 *C. difficile* strains. These strains were classified into three sequence types (STs), with ST11/RT12 being the most common [12]. In another study, Masarikova et al. isolated 44 *C. difficile* strains from 297 calf fecal samples. Of these strains, 84% were ST11 (RT033) [13]. A study in China also found that ST11 is the predominant ribotype in pigs [9]. These studies suggest that ST11 *C. difficile* strains are primarily hosted by livestock. We hypothesized that animal-associated strains can be transmitted to humans, which could be an important route of CDI transmission. However, further research is needed to characterize the genotypes and virulence phenotypes of ST11 strains isolated from humans.

With the fast development of sequencing technology, next-generation sequencing (NGS) based on the Illumina NovaSeq platform has enabled us to obtain high-quality bacterial genomic data with more accuracy and speed. The accurate genomic sequence of CDI-causing *C. difficile* strain has extensively promoted the study of *C. difficile* gene function and pathogenesis. The well-known model strain of *C. difficile*, 630, which is virulent and multidrug-resistant, was first sequenced by Sebaihia et al. in 2006 [14]. It was subsequently reannotated by Dupuy [15] and Lawley [16]. Dannheim et al. manually curated the genome sequence of *C. difficile* strain 630∆*erm* (DSM 28645), as well as the genome sequence of *C. difficile* strain 630 (DSM 27543). They completely identified the sequence of the transposon Tn5397 [17]. The genome sequence of *C. difficile* LCL126, obtained from the Lanzhou Institute of Biological Product, was revealed, along with its encoded proteins and potential toxicological genes [18]. However, extensive research has been carried out on the epidemiology of CDI and molecular characteristics of CDI-causing *C. difficile* strains. However, there is a general lack of research in the detailed genomic characterization of ST11 *C. difficile* strains isolated from CDI patients [19].

In the present study, we combined the NGS based on the Illumina NovaSeq platform and third-generation gene sequencing based on the Oxford Nanopore Technologies (ONT) platform to sequence a novel ribotype ST11 strain of *C. difficile* (ST11, PR34365, denoted WXL8) isolated from a CDI patient in the intensive care unit (ICU) of Affiliated Hospital of Guizhou Medical University. Comparative genomic sequence analyses with model strains were also performed to reveal the differences between typical *C. difficile* strain and WXL8.

## 2 Materials and methods

### 2.1 Isolation of *C. difficile* WXL8 and retrospective clinical data

Anal swab samples were obtained from patients at the Department of Critical Care Medicine, Affiliated Hospital of Guizhou Medical University in Guiyang, Guizhou, China. Samples were collected using sterile containers and transported under appropriate conditions to prevent contamination and degradation. Swab samples were then soaked in 2 mL of brain heart infusion medium (Solarbio, Lot. 328U032, Beijing, China) for 10 min and -mixed well. A portion of this sample was plated onto Cycloserine-Cefoxitin-Fructose Agar (CCFA), which inhibits the growth of competing flora while promoting the growth of *C. difficile*, plated and incubated at 37°C within an anaerobic chamber (N_2_: 95%, H_2_: 5%) for 48–72 h. Colonies suspected of *C. difficile* were identified based on their morphology and color on the selective media (light yellow, irregular edges, translucence). They underwent 16S rDNA amplification and sequencing by Sangon Biotech (Shanghai, Co., Ltd, China). The BLAST algorithm [20,21] was employed to classify the isolate taxonomically. Medical records from the Affiliated Hospital of Guizhou Medical University were acquired with patient consent, specifically detailing inpatient treatment and antibiotic usage history.


**Informed consent:** Informed consent has been obtained from all individuals included in this study.
**Ethical approval:** The research related to human use has been complied with all the relevant national regulations, institutional policies and in accordance with the tenets of the Helsinki Declaration, and has been approved by the Ethics committee of Guizhou Medical University (Approval no.: 2021069).

### 2.2 DNA extraction and genome sequencing

The *C. difficile* WXL8 strain’s genomic DNA was extracted using the sodium dodecyl sulfate method [22]. The extraction process involved cell lysis, removal of contaminants, and purification of the DNA. The quality and quantity of the extracted DNA were assessed using spectrophotometry and gel electrophoresis to ensure that the DNA was suitable for sequencing. Subsequently, the concentration of the genomic DNA was quantified by applying the Quant-iT PicoGreen dsDNA Assay Kit and the NanoDrop Spectrophotometer (Thermo Scientific, Waltham, USA). Following the successful qualification of the samples, DNA libraries were meticulously constructed. The Whole Genome Shotgun approach was employed to sequence the whole genome of the *C. difficile* WXL8 strain. Notably, the libraries were subjected to separate sequencing runs utilizing both NGS (Illumina NovaSeq, United States of America, California, NovaSeq 6000) and third-generation single-molecule sequencing technologies (ONT) platform.

### 2.3 Genome assembly and visualization

The raw sequencing data were processed using bioinformatics tools to assemble the genomes, annotate genes, and identify genetic features relevant to virulence and antibiotic resistance. Raw paired-end sequencing data were initially archived in the FASTQ format (Illumina 1.8+). The assessment of sequencing data quality was carried out using FastQC software [23]. Subsequently, the removal of 3′ adapter contamination was executed through the application of adapter removal [24]. Data underwent filtration based on Kmer frequency (Kmer = 17) using SOAPec (v.2.03) [25], resulting in the extraction of high-quality data from second-generation sequencing, which we refer to as HQdata. Canu software (v1.7.1) [26] was instrumental in assembling the triple sequencing data derived from the ONT platform (Oxford Nanopore Technologies Limited, Oxford, England). Following assembly, all resultant sequences were integrated to construct a comprehensive genome sequence, a process culminating in genome refinement through the utilization of Pilon software [27].

For third-generation sequencing data, assembly into contigs was achieved by employing HGAP [28] and CANU [26]. The HQdata was used to rectify the contigs using Pilon software (v.18) [27]. Finally, the obtained genome sequence, gene prediction, and non-coding RNA prediction information were integrated into a standard GBK (GenBank) format file, and the complete genome of *C. difficile* WXL8 was assembled using Circos software with predicted condensed proteins for condensed sequences. A circular map of this genome was then drawn using Cgview [29].

### 2.4 Gene function annotation

Gene prediction was performed employing GeneMarkS software (version 4.32, Georgia Institute of Technology, Atlanta, Georgia, USA), and the resulting amino acid sequences were subjected to a BLAST search against various databases, including gene ontology (GO), Kyoto Encyclopedia of Genes and Genomes (KEGG), clusters of orthologous groups of proteins (COG), carbohydrate-active enzymes database (CAZy), and the pathogen–host interactions database (PHI). Subsequently, a comprehensive filtering and annotation process was implemented.

### 2.5 Scanning electron microscopy (SEM) and growth curve determination

*C. difficile* was cultivated in a brain heart infusion-supplemented (BHIS) medium until reaching an optical density at 600 nm (OD_600_) of 0.6. Subsequently, the bacterial culture was harvested via centrifugation at 3,440 × g for 3 min. The collected bacteria were resuspended in a 2.5% glutaraldehyde fixative solution and left to fix overnight at 4°C. After fixation, the cells underwent a series of washes with phosphate-buffered saline (Gibco, Paisley, Scotland, UK), with each wash being repeated three times. Dehydration of the samples was then accomplished through sequential immersion in ethanol solutions with increasing concentrations: 50, 70, 90, and 100% (v/v), each for 5 min. The dehydrated samples were subsequently subjected to vacuum freeze-drying. Bacteria were delicately isolated from the specimens using a toothpick, affixed to an adhesive substrate, and sputter-coated with a gold film for SEM analysis, conducted with a Hitachi S-3400 SEM instrument (Japan). In parallel, seed *C. difficile* strain WXL8 strain was cultured to an OD_600_ of 0.5, then inoculated into BHIS liquid medium with a 1% inoculum and incubated anaerobically at 37°C. Optical density readings at OD_600_ were recorded at 3 h intervals, enabling the construction of growth curves.

### 2.6 Multilocus sequence typing (MLST) and capillary gel electrophoresis-based ribotyping

For MLST analysis, we selected housekeeping genes commonly used for *C. difficile* typing, including *adk*, *atpA*, *dxr*, *glyA*, *recA*, *sodA*, and *tpi* (Table 1). Specific primers for each selected housekeeping gene were designed based on previously published sequences [30]. PCR amplification was performed using a standard thermal cycler with the following conditions: initial denaturation at 95°C for 5 min, followed by 30 cycles of denaturation at 95°C for 30 s, annealing at 55°C for 30 s, and extension at 72°C for 1 min, concluding with a final extension at 72°C for 5 min. The PCR products were purified using a commercial purification kit (e.g., Qiagen QIAquick PCR Purification Kit) and then sequenced using Sanger sequencing technology. The resulting sequences were analyzed for quality and trimmed to remove low-quality bases. The sequences were compared to *C. difficile* MLST database (https://pubmlst.org/cdifficile//) to assign alleles and determine the ST of each isolate [9].

**Table 1:** MLST

Strain	*adk*	*atpA*	*dxr*	*glyA*	*recA*	*sodA*	*tpi*	ST types
WXL8	5	8	5	11	9	11	8	11

For ribotyping, the 16S–23S rDNA intergenic spacer region of WXL8 was amplified using the primers F1/R1 [31] and F2/R2 [32] (Table 5), which are labeled with 6-carboxyfluorescein (6-FAM) at the 5′ end. The reaction mixture was prepared as follows: 25 μL, including 2× Phanta Flash Master Mix (Dye Plus) (P520, Novozymes, Nanjing, China), 2 μL of each primer, 1 μL of WXL8 genomic DNA (200 ng/μL), and ddH_2_O to bring the total reaction volume to 25 μL. The PCR cycling conditions were as follows: 30 s of pre-denaturation at 98°C, 10 s of denaturation at 98°C, 5 s of annealing at 57°C, and 1 min of extension at 72°C for 35 cycles, and 1 min of final extension at 72°C. The PCR products were then separated by capillary electrophoresis performed at Sangon Biotech (Shanghai) Co., Ltd. The capillary electrophoresis results (Table 2) were submitted to the Webribo database (http://webribo.ages.at) for *C. difficile* ribotyping [33].

**Table 2:** Capillary gel electrophoresis-based ribotyping

Fragment a	Fragment b	Fragment c	Fragment d	Fragment e	Fragment f
308.02 bp	324.77 bp	327.23 bp	345.33 bp	370.66 bp	443.29 bp

### 2.7 Construction of phylogenetic tree

Seventy-nine *C. difficile* strains, including WXL8, were selected for phylogenetic analysis by reference to Dingle et al. [34]. The core genome of 79 strains was identified by clustering analysis using Pirate v1.0.4. Multiple sequence alignment was performed using mafft v7.475, and phylogenetic analysis was conducted using MEGA (MEGA X, Mega Limited, Auckland, New Zealand) to construct a maximum likelihood tree.

### 2.8 Comparative genomic analysis

In this study, we sought to elucidate the evolutionary trajectory of *C. difficile* strain WXL8. To achieve this, we retrieved the whole genome sequences of four reference strains, namely *C. difficile* CD630 (CD630), *C. difficile* CDS-0253, *C. difficile* CDR20291, and *C. difficile* CDBR81, from the National Center for Biotechnology Information. The Mauve software (version 2.4.0, http://darlinglab.org/mauve/mauve.html) was employed to facilitate a comprehensive comparative analysis of the genome of *C. difficile* WXL8 and these four reference *C. difficile* strains. Furthermore, Venn plot was generated using R package VennDiagram and Genome Collinearity Chord Diagram was generated using the chord-Diagram function from R package “circlize” (v.0.4.8).

## 3 Results

### 3.1 Retrospective clinical data of the host of *C. difficile* WXL8

A 53-year-old male patient was admitted to the ICU at Guizhou Medical University, presenting with an anterior communicating artery aneurysm and concomitant lung infection. Throughout his hospitalization, the patient underwent an initial therapeutic regimen consisting primarily of glycylcycline and β-lactam antibiotics, administered for 21 days. Notably, after 8 days of antibiotic treatment, the patient experienced the onset of mild diarrhea, prompting the collection of a stool sample via anal swab for further analysis. Subsequent culture of the stool sample on a CCFA plate yielded a distinctive colony exhibiting typical characteristics of *C. difficile*, characterized by its light-yellow hue, irregular border, and translucency. Subsequent analysis via BLAST (version 2.13.0) revealed a strikingly close genetic affinity between this strain and *C. difficile* ATCC9689, with an impressive 99.97% sequence identity. Consequently, this isolate was designated as *C. difficile* WXL8.

### 3.2 Morphology and growth characteristics of WXL8

**Figure 1:**
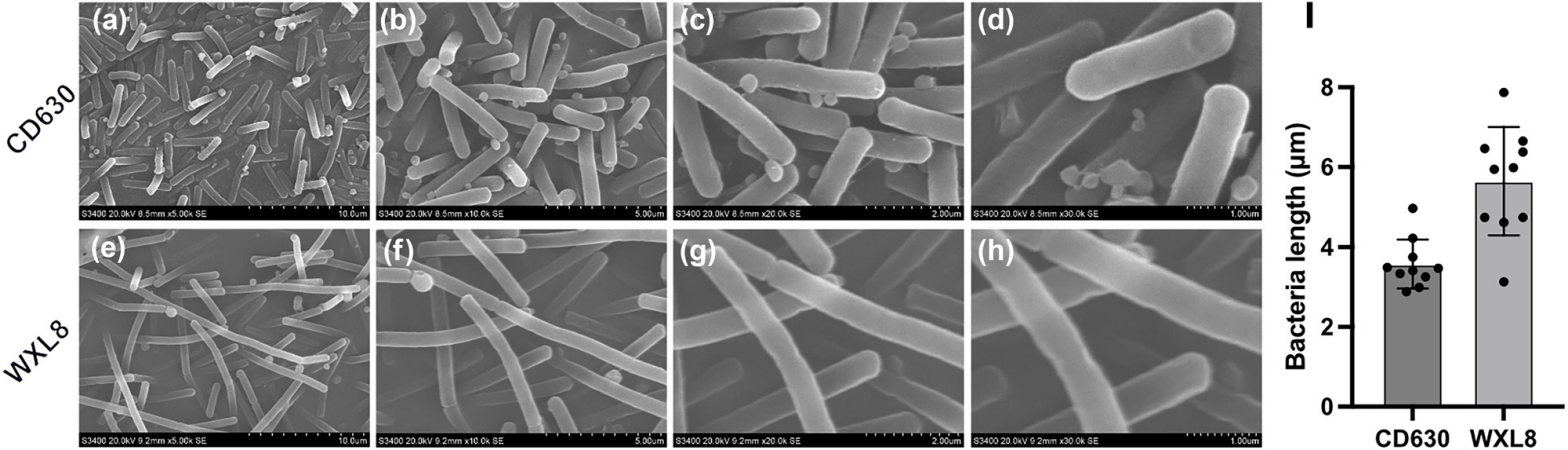
Observation of cell morphology of WXL8 via SEM. (a)–(d) Cell morphology of *C. difficile* CD630 at magnifications of 5,000 ×, 10,000 ×, 20,000 ×, and 30,000 ×, respectively. (e)–(h) Cell morphology of WXL8 at the same magnifications. Additionally, (i) the average length of WXL8 is compared to *C. difficile* CD630.

**Figure 2:**
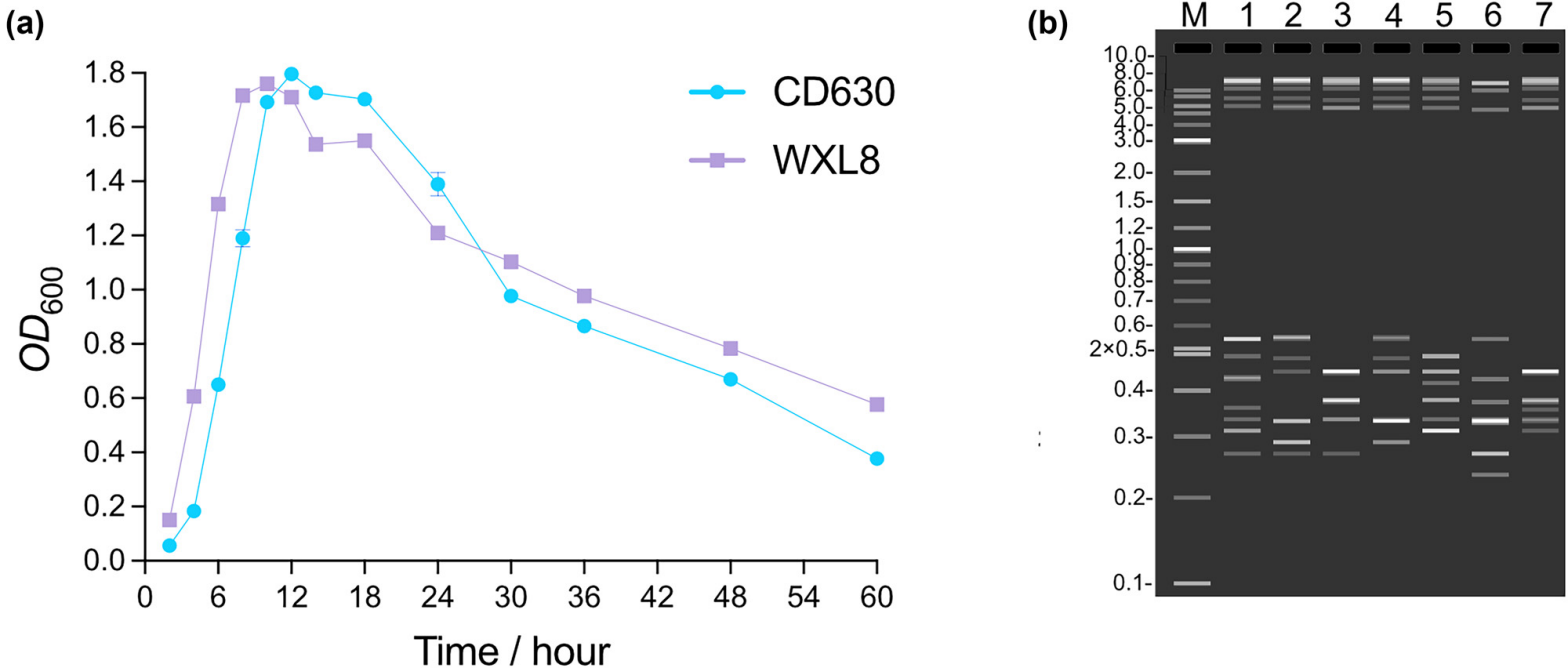
Growth curve and ribotyping analysis of WXL8. (a) The *x*-axis illustrates the culture time, while the *y*-axis represents the optical density (OD_600_) values measured at 600 nm. (b) A comparison between the electronic gel electrophoresis pattern of WXL8 and the well-known ST11 subribotypes is presented. (b) Virtual electrophoresis profiles of PCR ribotyping of seven *C. difficile* strains. M is a 1 kb Plus DNA Ladder (New England Biolabs). Lane 1 is CD630 (RT012), lane 2 is BR81 (RT106), lane 3 is DSM 101085 (RT033), lane 4 is S-0253 (RT002), lane 5 is M120 (RT078), lane 6 is R20291 (RT027), and lane 7 is WXL8.

The morphological attributes of both the *C. difficile* CD630 and WXL8 strains were observed using electron microscopy at magnifications of 5,000×, 10,000×, 20,000×, and 30,000×. The observations revealed a noteworthy distinction, with the bacterium of WXL8 displaying greater length when compared to CD630 (average length: 5.65 μm vs 3.58 μm), as illustrated in Figure 1a–i. Additionally, we assessed the growth profiles of WXL8, as depicted in Figure 2a. The growth rate of WXL8 exhibited a rapid increase, reaching its maximum growth concentration (OD_600_ = 1.76) at 10 h, which was slightly faster than the CD630 strain. Subsequently, at the 12 h, the onset of autolysis of WXL8 became evident, which was earlier than the CD630 strain. Virtual electrophoresis results using genomic DNAs as templates showed that WXL8 had a different ribotype compared to the six typical *C. difficile* strains: CD630 (RT012), BR81 (RT106), DSM 101085 (RT033), S-0253 (RT002), M120 (RT078), and R20291 (RT027) (Figure 2b).

### 3.3 Genome characteristics of *C. difficile* WXL8

**Figure 3:**
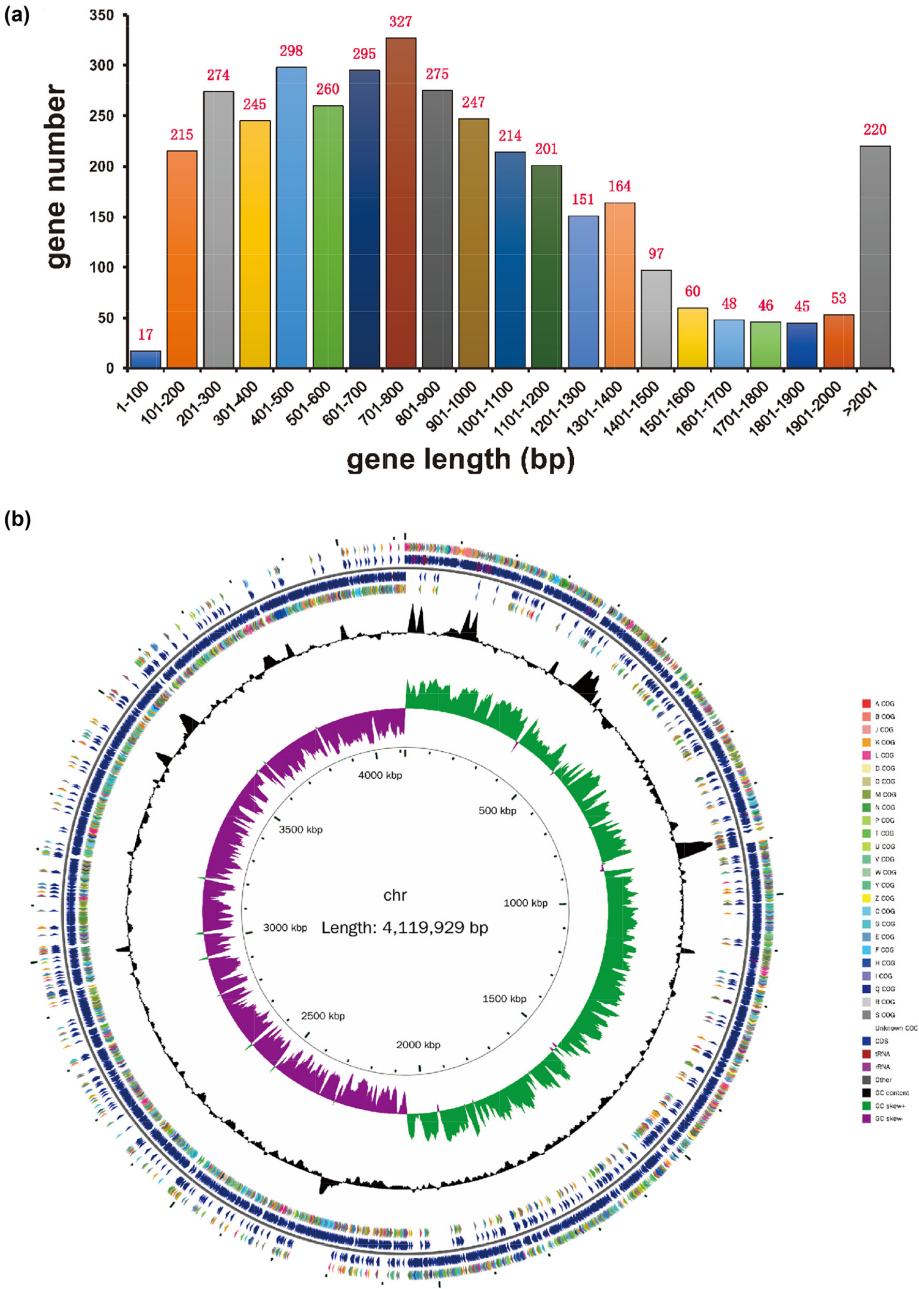
Gene length statistics and genome circle map of WXL8. (a) The *x*-axis represents gene length, while the *y*-axis shows the corresponding gene count. (b) In the circular representation, the innermost circle denotes the scale, followed by the depiction of GC Skew, GC content, and the COG of each CDS from the fourth to seventh circles. The fifth and sixth circles provide the positional information of CDS, tRNA, and rRNA within the genome.

High-throughput sequencing data provided comprehensive insights into the genomic characteristics of *C. difficile* WXL8. The circular genome of *C. difficile* WXL8 was determined to encompass a total size of 4,119,929 base pairs (bp) with a GC content of 29.06% (GenBank Accession Number: CP137339). Notably, our examination did not reveal the presence of any plasmids within its genome, as illustrated in Figure 3. The WXL8 genome contains a total of 3,752 predicted protein-coding genes, with an average length of 906.28 bp (accounting for 82.53% of the total length) (Figure 3a). To visualize the circular structure of the WXL8 genome, we utilized the cgview tool, and the graphical representation is presented in Figure 3b. The genomic content also encompassed a diverse repertoire of 271 RNA copies, including 35 rRNA copies (11 copies of 5S rRNA, 12 copies of 16S rRNA, and 23S rRNA), 89 tRNA genes, and 147 non-coding RNAs.

Additionally, we identified a total of 178 repetitive sequences, comprising 27 long interspersed nuclear elements, 88 long terminal repeats, 39 DNA transposons, 2 satellite RNAs, and 8 unclassified interspersed repeats. Our investigation also detected 16 prophages within the genome, collectively spanning a total length of 407,296 bp, with an average prophage length of 25,456 bp. Furthermore, we predicted the presence of 15 clustered regularly interspaced short palindromic repeats (CRISPRs) arrays, each characterized by an average length of 777.4 bp. Lastly, our analysis revealed the prediction of 33 genomic islands (GIs), with a cumulative length of 440,363 bp and an average length of 16,310 bp. Detailed information regarding the spatial distribution of these gene islands can be found in Figure S1.

### 3.4 MLST typing and ribotyping

The genome sequence of WXL8 was uploaded to the MLST database. As shown in Table 1, the results indicated that the MLST type of WXL8 was ST11, which is common in livestock but relatively rare in humans [35]. The ribotype identification results, as shown in Table 2, indicated that the WXL8 strain was defined as a novel ribotype, PR34365, by the Webribo database.

### 3.5 Genome functional analysis of *C. difficile* WXL8

**Figure 4:**
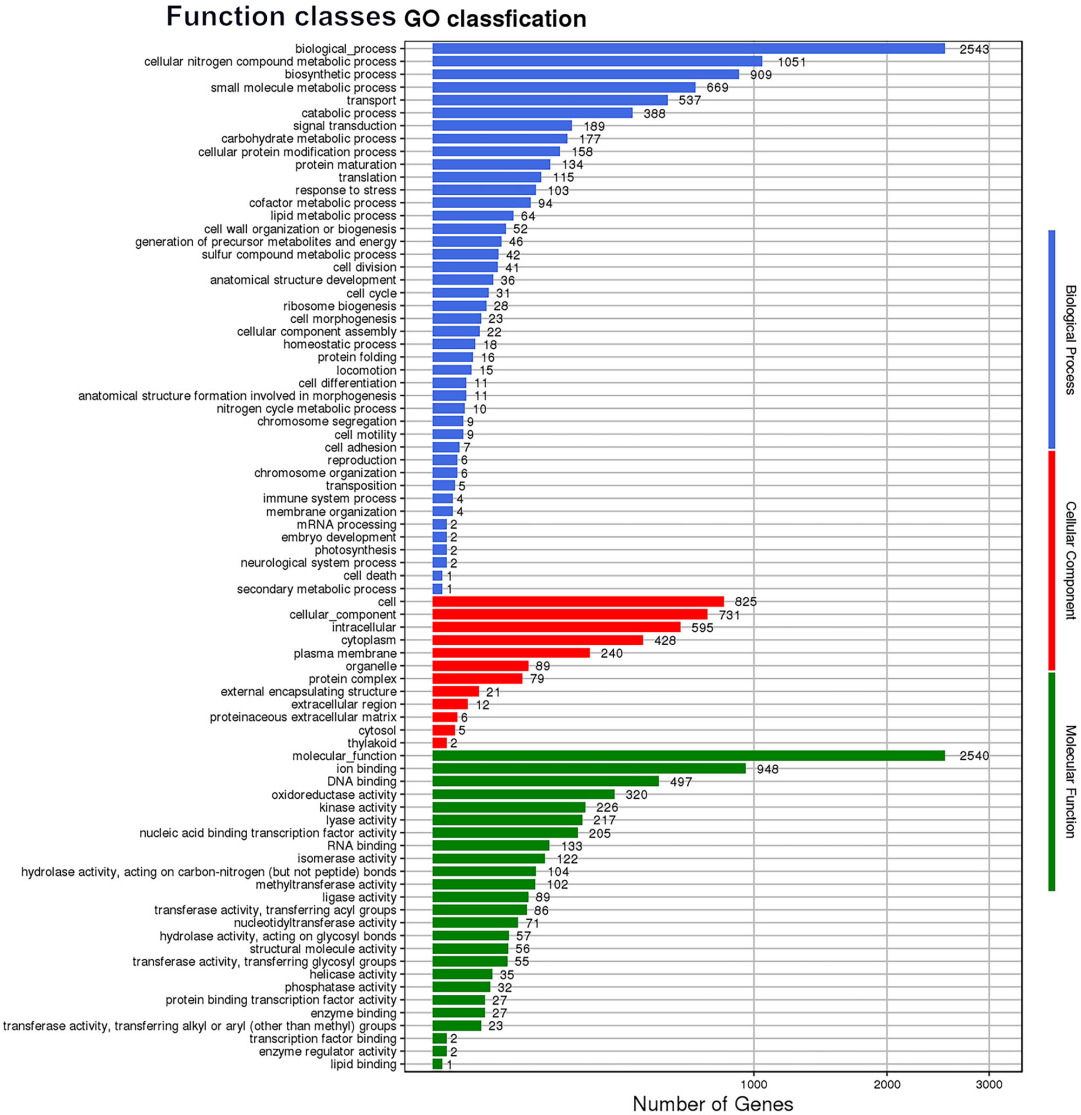
GO functional annotation across the entire genome of WXL8. The *x*-axis denotes the number of genes in the sample annotation, while the *y*-axis represents the GO functional classification.

To elucidate the functional aspects of the *C. difficile* WXL8 genome, a comprehensive investigation encompassing gene annotation, effector profiling, and virulence analysis was conducted. GO annotation yielded a substantial dataset, revealing a total of 16,603 functionally classified terms. Within this dataset, three primary ontology categories were identified: biological process (2,543 terms), cellular component (825 terms), and molecular function (2,540 terms), as depicted in Figure 4. These categorizations collectively constitute a comprehensive framework for comprehending the multifaceted gene functions within the genome of *C. difficile* WXL8.

**Figure 5:**
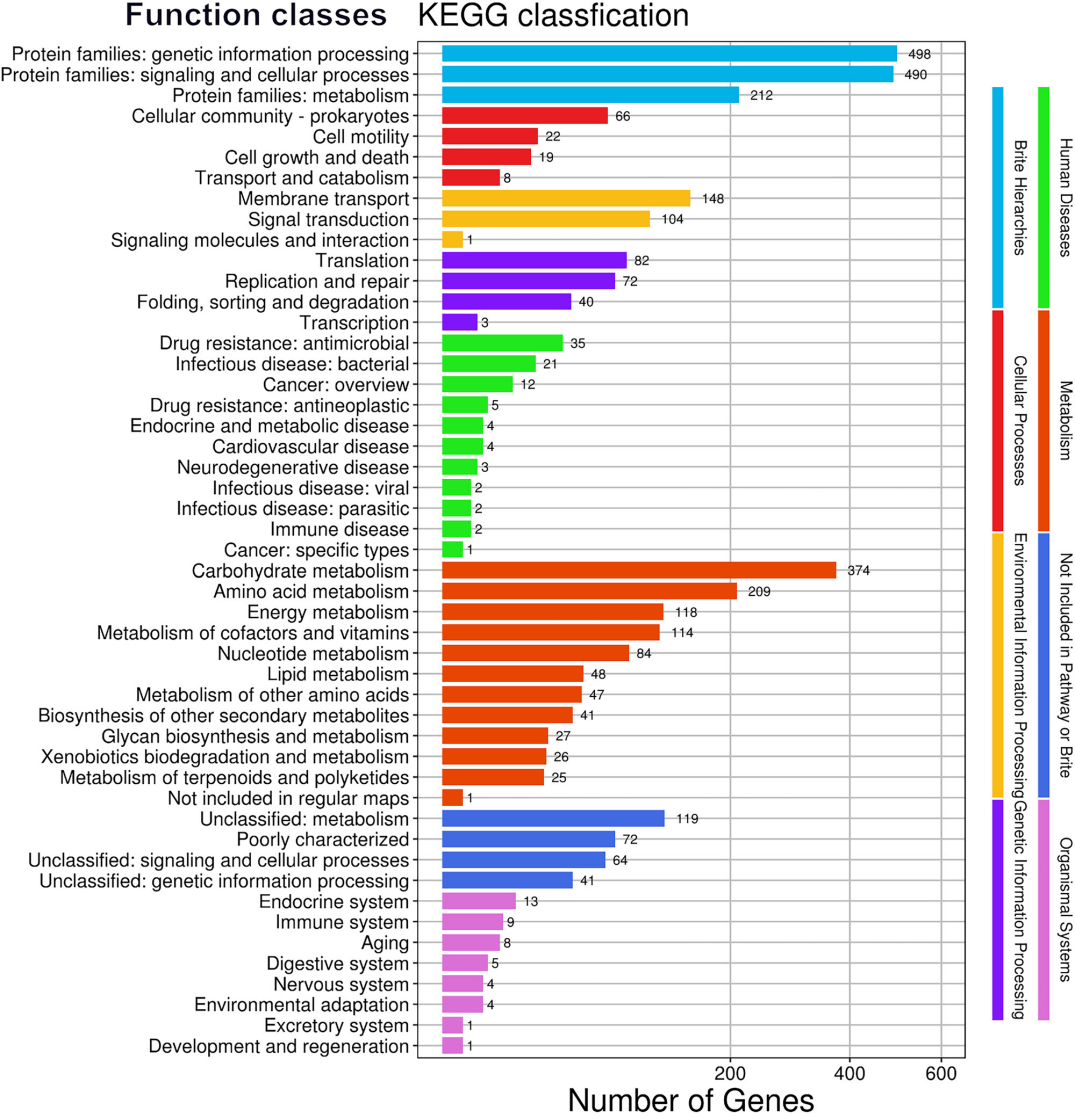
Distribution of genes based on the KEGG classification in WXL8. The numbers on the *x*-axis indicate the count of genes in the annotation, while the *y*-axis represents the distinct functional categories.

The gene function exploration of *C. difficile* WXL8 was further enriched through KEGG annotation, which revealed a comprehensive dataset comprising 3,311 genes. These genes were thoughtfully categorized into eight distinct pathways, delineated as Brite Hierarchies, Cellular Processes, Environmental Information Processing, Genetic Information Processing, Human Diseases, Metabolism, Not Included in Pathway or Brite, and Organismal Systems (Figure 5). Among these pathways, notable prominence was observed in various categories. In the Brite Hierarchies class, 498 unigenes were associated with protein families predominantly influencing genetic information processing. In the Cellular Processes class, 66 unigenes played pivotal roles in cellular community-prokaryotes, while the Environmental Information Processing class featured 148 unigenes notably associated with membrane transport. Within the Genetic Information Processing class, 82 unigenes held significance in the domain of translation, further enriching our understanding of this fundamental process. The Human Diseases class unveiled 35 unigenes contributing to drug resistance, particularly in the context of antimicrobial resistance. Metabolism, a critical aspect of cellular function, was notably represented by 374 unigenes in the metabolism class, with a predominant focus on carbohydrate metabolism. In the Not Included in Pathway or Brite class, 119 unigenes were identified, primarily associated with unclassified metabolism. Lastly, in the Organismal Systems class, 13 unigenes were intricately linked to the endocrine system, further expanding our comprehension of the genomic contributions to this intricate biological system. This comprehensive categorization provides a valuable framework for deciphering the multifaceted functional aspects of genes within the *C. difficile* WXL8 genome.

**Figure 6:**
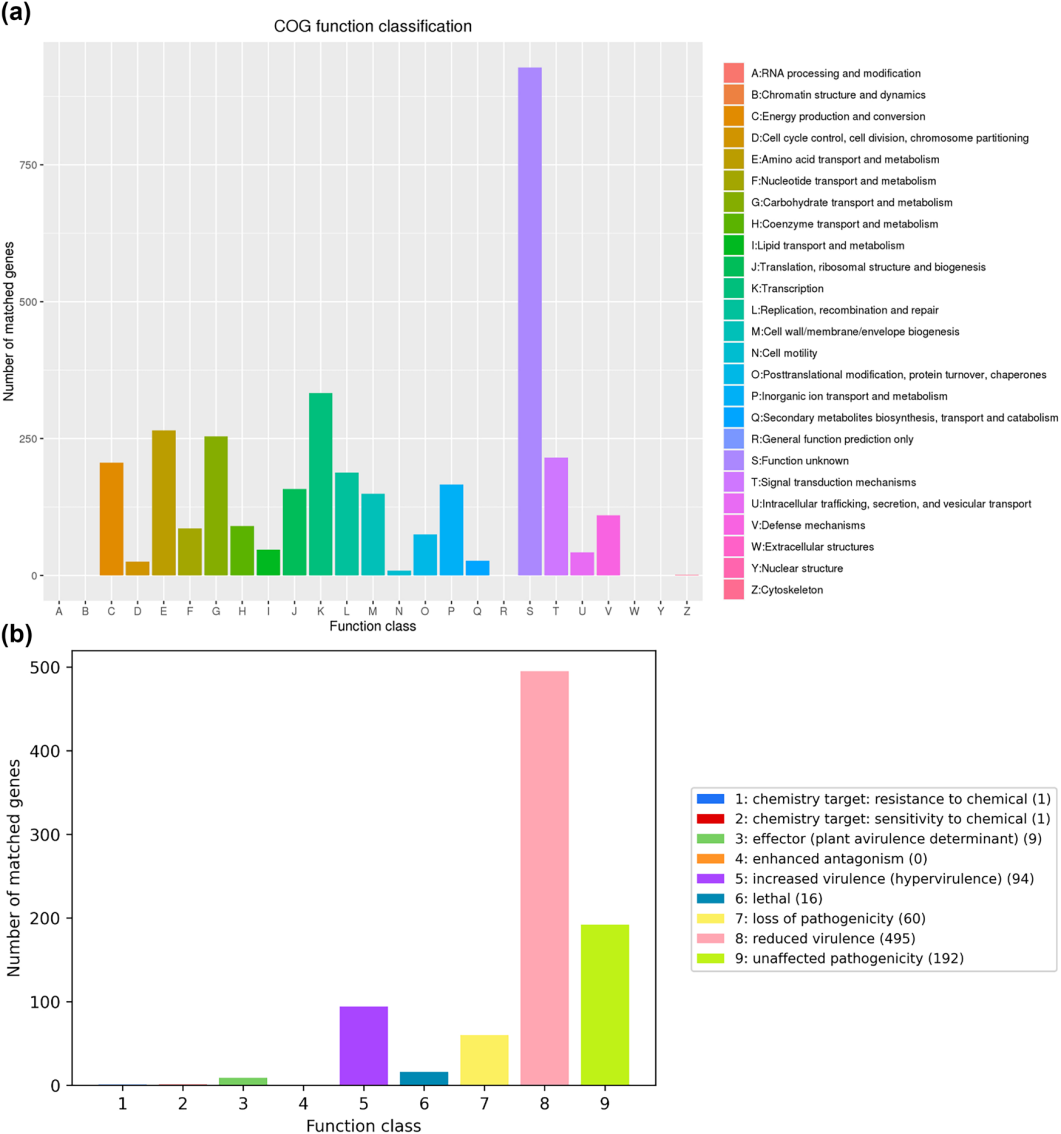
Annotated clusters based on the COG in WXL8 and the mutated genes in the PHI. (a) Functional classification is represented along the *x*-axis, while the count of genes is shown on the *y*-axis. (b) Function classes are indicated along the *x*-axis, and the number of mutated genes is represented on the *y*-axis.

**Figure 7:**
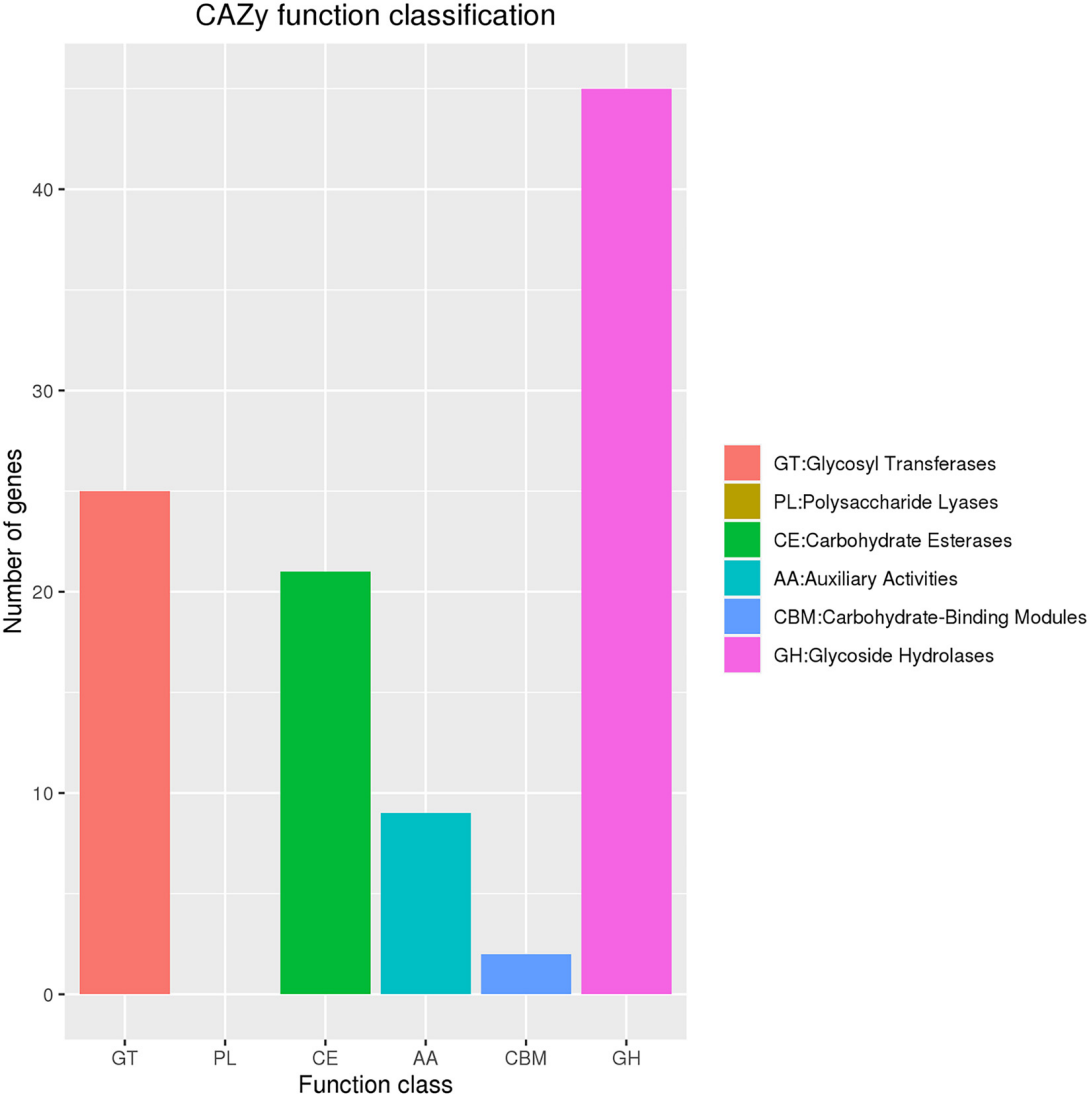
CAZy functional classification chart for WXL8. The *x*-axis denotes the number of genes in the sample annotation, while the *y*-axis represents the different CAZy functional categories.

In the COG database, we identified 3,374 genes and classified them into 24 distinct categories. The most abundant category was Transcription, which houses 333 genes, followed closely by Amino Acid Transport and Metabolism and Carbohydrate Transport and Metabolism, which included 265 and 254 genes, respectively (Figure 6a). Additionally, 69 genes were associated with defense mechanisms, while the functions of 928 genes remained uncharted. In the CAZy database, we annotated 102 genes, comprising 45 glycoside hydrolases, 25 glycosyl transferases, 21 carbohydrate esterases, 9 auxiliary activities, and 2 carbohydrate-binding modules (Figure 7). Furthermore, we identified 77 signal peptides, 961 transmembrane structures, and 94 secreted proteins. These secreted proteins are pivotal in the context of infection, where they play a crucial role in the intricate pathogen–host interactions that underlie CDI.

Analysis in the Virulence Factors of Pathogenic Bacteria (VFDB) database revealed that the *C. difficile* WXL8 strain carries nine toxin genes, including Toxin A, Toxin B, UDP-N-acetylglucosamine-2-epimerase, CdtA, CdtB, lota-toxin components Ia and Ib, along with two copies of ATP-dependent Clp protease proteolytic subunit (Table 3). Furthermore, the PHI examination indicated that 495 genes could potentially reduce toxicity, while 94 genes had the opposite effect (Figure 6b). In the Comprehensive Antibiotic Resistance Database (CARD) analysis, a total of 35 genes were annotated, encompassing 22 antibiotic resistance genes and 17 antibiotic target genes (Table 4). These findings shed light on the diverse genomic attributes of *C. difficile* WXL8, which contribute to its functional repertoire and interactions with its host environment.

**Table 3:** VFDB result statistics

VFDB ID	ORF name	VFDB name
VFG002288(gb|YP_001087135)	chr_689	VF0377
VFG002287(gb|YP_001087137)	chr_692	VF0376
VFG001373(gb|NP_344890)	chr_1022	VF0144
VFG002298(gb|AAF81760)	chr_2636	VF0385
VFG002292(gb|CAA51959)	chr_2636	VF0381
VFG002299(gb|AAF81761)	chr_2637	VF0385
VFG002293(gb|CAA51960)	chr_2637	VF0381
VFG000077(gb|NP_465991)	chr_3370	VF0074
VFG000077(gb|NP_465991)	chr_3395	VF0074

**Table 4:** Statistics of antibiotic resistance analysis results

Seq ID	Property	Number of genes	Percentage
chr	Antibiotic resistance	22	0.586
	Antibiotic target	17	0.453
	Antibiotic biosynthesis	0	0.000
	Total genes	35	0.933

### 3.6 Phylogenetic analysis

**Figure 8:**
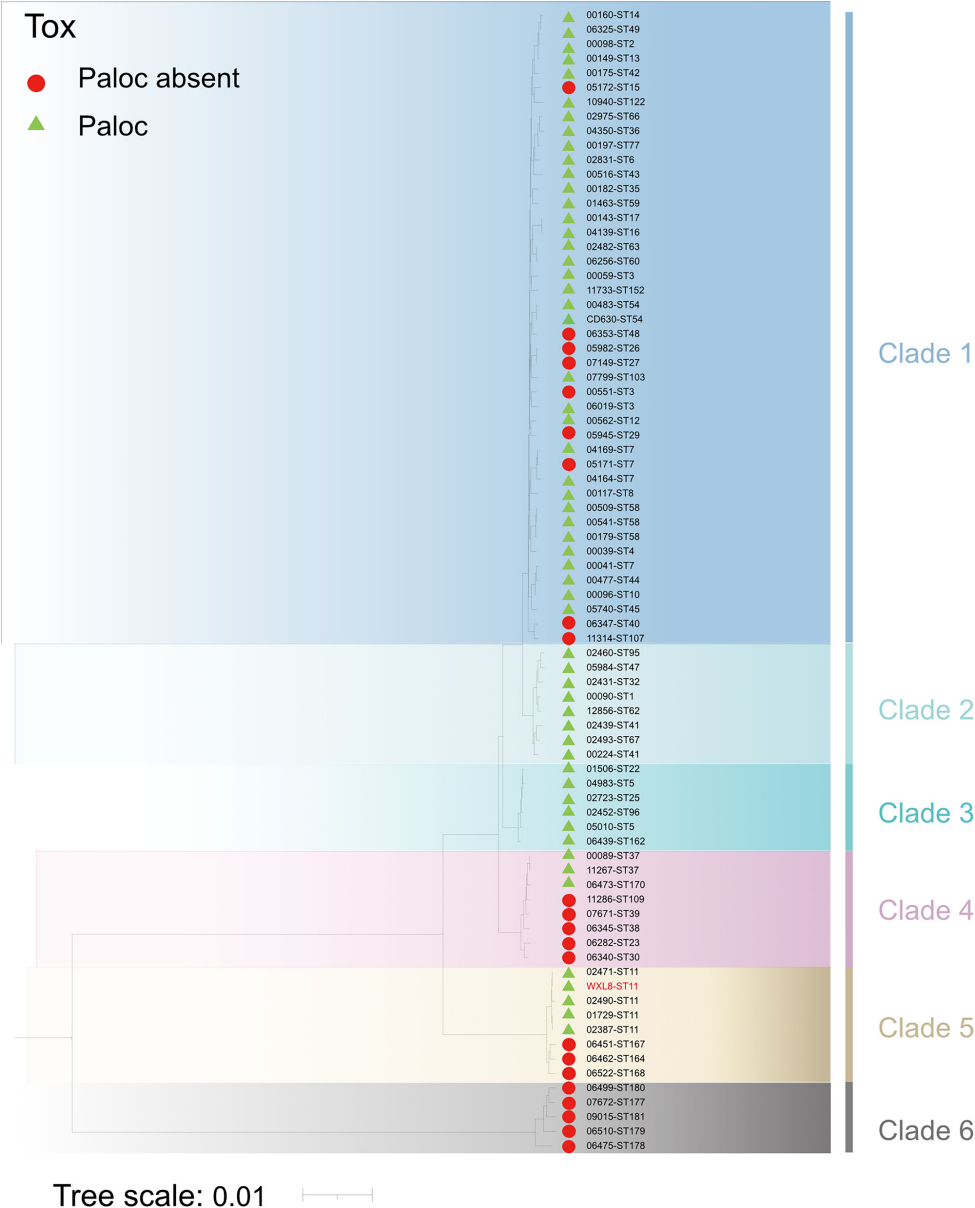
Phylogenetic analysis. The evolutionary relationships of WXL8 and typical *C. difficile* strains were depicted by a phylogenetic tree using the Neighbor-Joining method. Green triangles denote virulence-producing strains, while non-virulence-producing strains are marked with red circles. Different clades are depicted in different colors. The scale of the tree is 0.01.

To explore the phylogenetic status of WXL8, we constructed a phylogenetic tree that incorporated WXL8 using the neighbor-joining method. The resulting phylogenetic tree showcased representatives of *C. difficile* species from the six recognized evolutionary branches and illuminated relationships between virulence-producing and non-virulence-producing isolates. In Figure 8, WXL8, along with four ST11 strains as well as ST164, ST167, and ST168, cluster in clade 5, holding a unique position in the evolutionary tree. Notably, all ST11 strains within this branch exhibit the *tcdA* ^+^ and *tcdB* ^+^ phenotype.

### 3.7 Comparative genomic analyses

**Figure 9:**
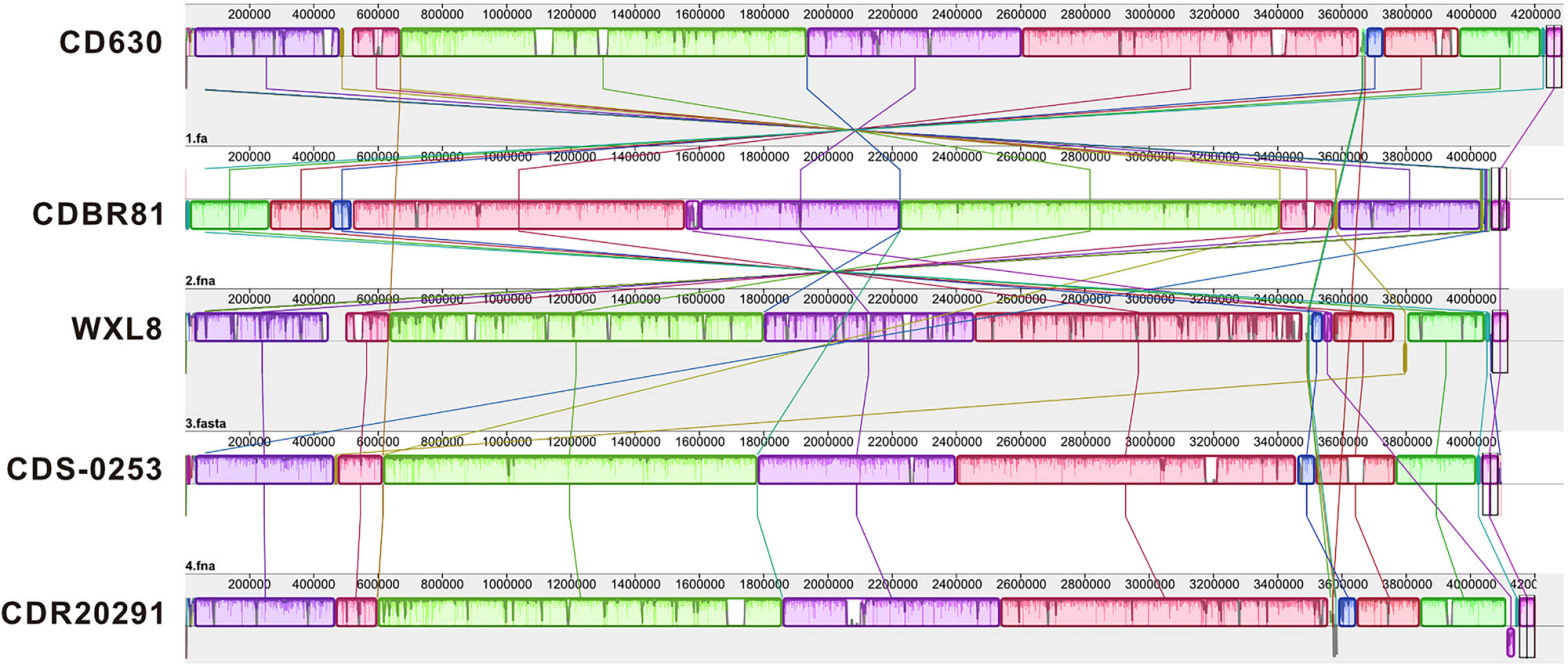
Multi-gene sequence comparison of strains WXL8, CD630, CDS-0253, CDR20291, and CDBR81. Mauve alignment of four representative complete genomes within each of the four species of *C. difficile*: CD630, CDBR81, WXL8, CDS-0253, and CDR20291. Blocks of the same color correspond to locally collinear blocks. Each sequence of identically colored blocks represents a collinear set of matching regions. One connecting line is drawn per collinear block.

Through collinear gene comparisons, we meticulously examined the consistency and variability among the whole genomes of *C. difficile* CD630 [14], CDBR81 [36], CDS-0253 [37], CDR20291 [38], and WXL8 strains, offering insights into their shared genetic ancestry. The comprehensive gene covariance analysis, as illustrated in Figure 9, enabled a closer inspection of WXL8 in comparison to the other four *C. difficile* strains. In terms of gene structure alignment, WXL8 exhibited a higher similarity to the reference genomes *C. difficile* CD630 and *C. difficile* CDR20291. Specifically, the gene structure arrangement in WXL8 closely resembled that of CDR20291, although notable distinctions were observed compared to *C. difficile* CDBR81.

The pan-genome encompasses all genes within a species. It comprises three main components: core genes (present in all strains), dispensable genes (not essential but present in some strains), and strain-specific unique genes [39]. We examined the genomic distribution of five *C. difficile* strains – 630, R20291, S-0253, BR81, and WXL8 – using a Venn diagram (Figure S2a) to determine the number of core and unique genes across samples. The results revealed that WXL8 contains 4,372 genes, with 3,096 shared with the strains 630, R20291, S-0253, and BR81. Notably, *C. difficile* strain WXL8 possesses the highest number of unique genes (253) among the five examined strains. The R20291 strain has 155 unique genes, while strain 630 has 123, strain S-0253 contains 74, and strain BR81 exhibits the fewest, with only 53 unique genes. This indicates that WXL8 displays a distinct genomic profile compared to other strains.

Synteny refers to the conservation of gene order on chromosomes across different species. Generally, more distantly related species exhibit less gene synteny, thus serving as a metric for evolutionary distance. We conducted a synteny analysis between the genomes of *C. difficile* 630 and WXL8 to explore their genomic relationships. The results are depicted in Figure S2b, where colored lines illustrate connections between the two genomes; blue represents *C. difficile* 630, while orange denotes *C. difficile* WXL8. This result representation effectively highlights the corresponding genomic regions between WXL8 and *C. difficile* strain 630. Overall, WXL8 exhibits similarity to the reference genomes of *C. difficile* CD630 (Figure S2b).

A partial deletion was observed within the 0.4–0.6 Mb range of WXL8 compared to the other four genomes analyzed. In contrast to CD630, WXL8 exhibited a single reverse fragment insertion spanning 2.2–2.4 Mb and a double fragment insertion between 3.6 and 3.8 Mb. Interestingly, one fragment displayed a forward orientation while the other was reversed, contributing to genomic diversity. Additionally, an inversion event spanning 3.4–3.6 Mb was identified in WXL8 compared to CD630. Further investigation revealed inversions at the 2.2–2.4 Mb locus relative to the CDBR81 genome and fragment inversions within the 3.6–3.8 Mb region compared to the *C. difficile* CDR20291 genome. These comparative genomic analyses highlight the subtle variations and structural rearrangements that distinguish WXL8 from its closely related counterparts.

## 4 Discussion

*C. difficile* toxin is a major pathogenic factor associated with the occurrence of CDI in hospitals [40]. However, *C. difficile* can be isolated not only in human feces but also in a variety of animals (pigs, cattle, chickens, ducks, etc.), and with the emergence of highly virulent strains of *C. difficile*, CDI has now become a significant public health problem internationally [41]. In the present study, a strain of *C. difficile*, named WXL8 (PR34365), isolated from the feces of patients in the ICU ward of the Affiliated Hospital of Guizhou Medical University, was observed morphologically by SEM and its growth curve was measured. The whole genome sequence of WXL8 (PR34365) was obtained by high-throughput sequencing. The MLST typing and capillary gel electrophoresis-based ribotyping categorized it as ST11 genotype and PR34365 ribotype. A phylogenetic tree was constructed after extracting core genes, and comparative genomic analysis was performed with other *C. difficile* strains. The VFDB annotation results showed that WXL8 contained TcdA, TcdB, and the binary toxin CdtA/CdtB. In addition, 94 genes showed increased virulence in the PHI annotation results and 22 antibiotic resistance genes in the CARD annotation results. To our limited knowledge, the WXL8 is the first ST11(PR34365) *C. difficile* strain isolated from a CDI patient.

*C. difficile* sequence type 11 (ST11) is recognized for its global prevalence in infecting and colonizing livestock. In 2005, Goorhuis et al. first reported the isolation of human ST11 *C. difficile* [42]. It encompasses a diverse array of ribotypes, several of which are implicated in human diseases. This pattern indicates that CDIs could potentially be a zoonosis [43]. ST11 interests researchers as one of the world’s most widespread and threatening classes of genotypes, especially in Asia [44]. *C. difficile* RT078, a notable sub-lineage within ST11, has established significant reservoirs in livestock globally [45]. It has also been detected in various retail meat products across North America and Europe [46]. It is a leading cause of both hospital-acquired and community-acquired CDI in these regions [42,47,48]. However, previous studies have shown that ST11 CDI cases are rarely detected in China [49]. In 2021, Zhang et al. isolated 99 strains of *C. difficile* from diarrhea patients under 5 years of age, with the main genotypes being ST3, ST54, and ST35, and only one strain was ST11/RT078 (1%) [50]. The same research group isolated 116 strains of *C. difficile* from elderly fecal samples in 2018, of which 14 were ST11 (12%). In the study by Wang et al., 46 strains of *C. difficile* were isolated, with the primary genotype being ST54, and no ST11 strain was isolated [51]. The transmission route and epidemiological characteristics of the ST11 ribotype in CDI patients in China are still unclear [52]. This study suggests that ST11, which is common in livestock, can infect humans, confirming its zoonotic nature. Therefore, people who come into contact with livestock should be aware of the risk of CDI.

WXL8 was typed by MLST and capillary gel electrophoresis-based ribotyping and was known to belong to ST11(PR34365). The core genome of WXL8 was extracted, and the phylogenetic tree was constructed with 78 other *C. difficile* individuals. The results show that WXL8 is located on clade 5, a unique branch and that strains on this branch are mostly ST11 genotypes and present toxin genes. In China, Clade 1 is the dominant group of clinical isolates, while Clade 5 is relatively rare [53,54]. The virtual and capillary gel electrophoresis results showed that WXL8 had a unique ribotype. To be cautious in identifying the new ribotype, we repeated the analysis using two sets of primers (F1/R1, F2/R2, Table 5) labeled with 6-FAM. The results of both sets of primers were consistent with the PR34365 ribotype. This ribotype was first reported in ST11, and its clinical epidemiology needs to be further studied in future work.

**Table 5:** Strains and primers

Strain/primer	Relevant characteristic	Source
*C. diffcile* 630(AM180355.1)	4.29 Mb, 3,981 genes, 29.0% G + C content	Switzerland
*C. difficile* BR81(CP019870.1)	4.12 Mb, 3,747 genes, 28.5% G + C content	Korea
*C. difficile* s-0253(CP076401.1)	4.08 Mb, 3,732 genes, 28.5% G + C content	Australia
*C. difficile* R20291(CP029423.1)	4.20 Mb, 3,843 genes, 29.0% G + C content	United Kingdom
*C. difficile* WXL8(CP137339)	4.11 Mb, 3,752 genes, 29.0% G + C content	China
F1	5′-GTGCGGCTGGATCACCTCCT-3′	[31]
R1	5′-CCCTGCACCCTTAATAACTTGACC-3′	
F2	5′-GCTGGATCACCTCCTTTCTAAG-3′	[32]
R2	5′-TGACCAGTTAAAAAGGTTTGATAGATT-3′	

The comparative genomic analysis indicated that WXL8 has a unique gene arrangement compared to other strains such as CD630, CDS-0253, CDR20291, and CDBR81. The genomic analysis revealed that WXL8 harbors nine toxin genes (Table 3), including Toxin A (TcdA), Toxin B (TcdB), and the binary toxin genes (CdtA and CdtB). These toxins are critical for the pathogenicity of *C. difficile*, as they disrupt the host’s intestinal epithelial cells, leading to inflammation and diarrhea. The presence of these toxins suggests that WXL8 has the potential to cause severe disease, similar to other hypervirulent strains like RT027 and RT078. For instance, the presence of both *tcdA* and *tcdB* suggests a potential for severe disease manifestation, while the binary toxin (*cdtA*/*cdtB*) is associated with increased virulence and has been implicated in more severe clinical outcomes [55]. The CARD analysis revealed that WXL8 carries 22 antibiotic resistance genes and 17 antibiotic target genes, suggesting that WXL8 has a robust arsenal of resistance mechanisms. These genes could confer resistance to multiple antibiotics, including those commonly used to treat CDIs, such as metronidazole and vancomycin. The 15 CRISPR arrays identified in the WXL8 genome may play a role in antibiotic resistance by providing a defense mechanism against foreign genetic elements, such as plasmids carrying antibiotic resistance genes. This could allow WXL8 to maintain its resistance profile and potentially acquire new resistance genes through horizontal gene transfer. The presence of these resistance genes may explain the high recurrence rate of CDI in patients, as antibiotic pressure could be selected for resistant strains like WXL8.

Concern host interaction, first, the genome of WXL8 encodes 94 secreted proteins, which are crucial for host–pathogen interactions. These proteins may include enzymes that degrade host tissues, immune modulators that evade the host’s immune response, or adhesins that facilitate colonization of the intestinal mucosa. The presence of these secreted proteins suggests that WXL8 has evolved mechanisms to interact with and manipulate the host environment, enhancing its ability to cause infection. Second, the KEGG annotation revealed that WXL8 has a significant number of genes involved in carbohydrate metabolism, which could be crucial for its survival in the gut environment. The ability to efficiently metabolize various carbohydrates may give WXL8 a competitive advantage in the gut microbiome, allowing it to outcompete other bacteria and establish infection. Moreover, the identification of 33 GIs in WXL8, some of which may carry virulence-related genes, could contribute to its pathogenicity. These GIs might encode additional virulence factors or regulatory elements that enhance the strain’s ability to colonize and infect the host.

Identifying ribotype ST11(PR34365) may indicate a potential reservoir in animal populations, possibly a source of human infection. Understanding the host range of this ribotype is crucial for assessing its zoonotic potential. Investigating which animal species carry this ribotype can help identify potential reservoirs; for instance, if ST11(PR34365) is found in livestock, wildlife, or companion animals, it raises concerns about human transmission routes, especially in settings where humans and animals interact closely. The study of environmental and ecological factors that facilitate the transmission of ST11(PR34365) from animals to humans is essential, including examining food production systems, handling practices, and environmental contamination. The emergence of a novel ribotype with zoonotic potential can also have significant public health implications, necessitating enhanced surveillance in both animal and human populations. Monitoring for this ribotype in clinical settings can help in the early detection of zoonotic infections. Understanding the transmission dynamics of ST11(PR34365) can inform infection control measures in healthcare settings, particularly in hospitals where patients may be at higher risk of infection from zoonotic pathogens. Further conducting epidemiological studies to track the incidence of infections associated with ST11(PR34365) can help establish its role in zoonotic transmission while investigating the ecological niches and environmental conditions that favor the persistence and spread of ST11(PR34365) and can inform strategies to mitigate its impact. Continued research in this area is essential to safeguard public health and enhance our understanding of the complex interactions among humans, animals, and the environment.

Guizhou is a predominantly rural province with a significant agricultural sector, including livestock farming. The close interaction between humans and animals in this region increases the risk of zoonotic transmission of *C. difficile*. Public health interventions should be tailored to the specific needs of rural communities, including education on the risks of zoonotic diseases and the promotion of safe farming practices. The healthcare infrastructure in Guizhou, particularly in rural areas, may be less equipped to handle outbreaks of CDI. Strengthening healthcare capacity, including access to diagnostic tools and effective treatments, is essential to mitigate the impact of zoonotic *C. difficile* transmission. In healthcare settings, particularly in rural hospitals in Guizhou, infection control measures should be strengthened to prevent the spread of *C. difficile*. This includes proper hand hygiene, environmental cleaning, and the judicious use of antibiotics to reduce the risk of CDI. Public health campaigns should also educate farmers and the general public of Guizhou, China, about the risks of zoonotic transmission and the importance of good hygiene practices.

The findings of this study on the novel ST11(PR34365) *C. difficile* strain WXL8 both align with and differ from previous research on ST11 and other ribotypes. While the association with livestock, virulence factors, and antibiotic resistance are consistent with previous studies, identifying a novel ribotype and isolating a human patient in China provide new insights into the genetic diversity and zoonotic potential of the ST11 lineage. These findings underscore the importance of continued surveillance and research to understand the epidemiology, virulence, and resistance profiles of *C. difficile* strains, particularly in regions with close human–animal interaction. This study enriched the genomic data on *C. difficile* and laid the foundation for future exploration of the molecular epidemiology of *C. difficile* in Guizhou, China.

## Supplementary Material

Supplementary Figure
