# Supplementary material for: Genomic analysis of a novel ST11(PR34365) Clostridioides difficile strain isolated from the human fecal of a CDI patient in Guizhou, China
Source: Open Life Sci. 2025 May 20;20(1):20251067. doi: 10.1515/biol-2025-1067 (PMC12103185; doi:10.1515/biol-2025-1067)
Supplement: Supplementary Figure [file biol-2025-1067-sm.pdf]

Supplementary material

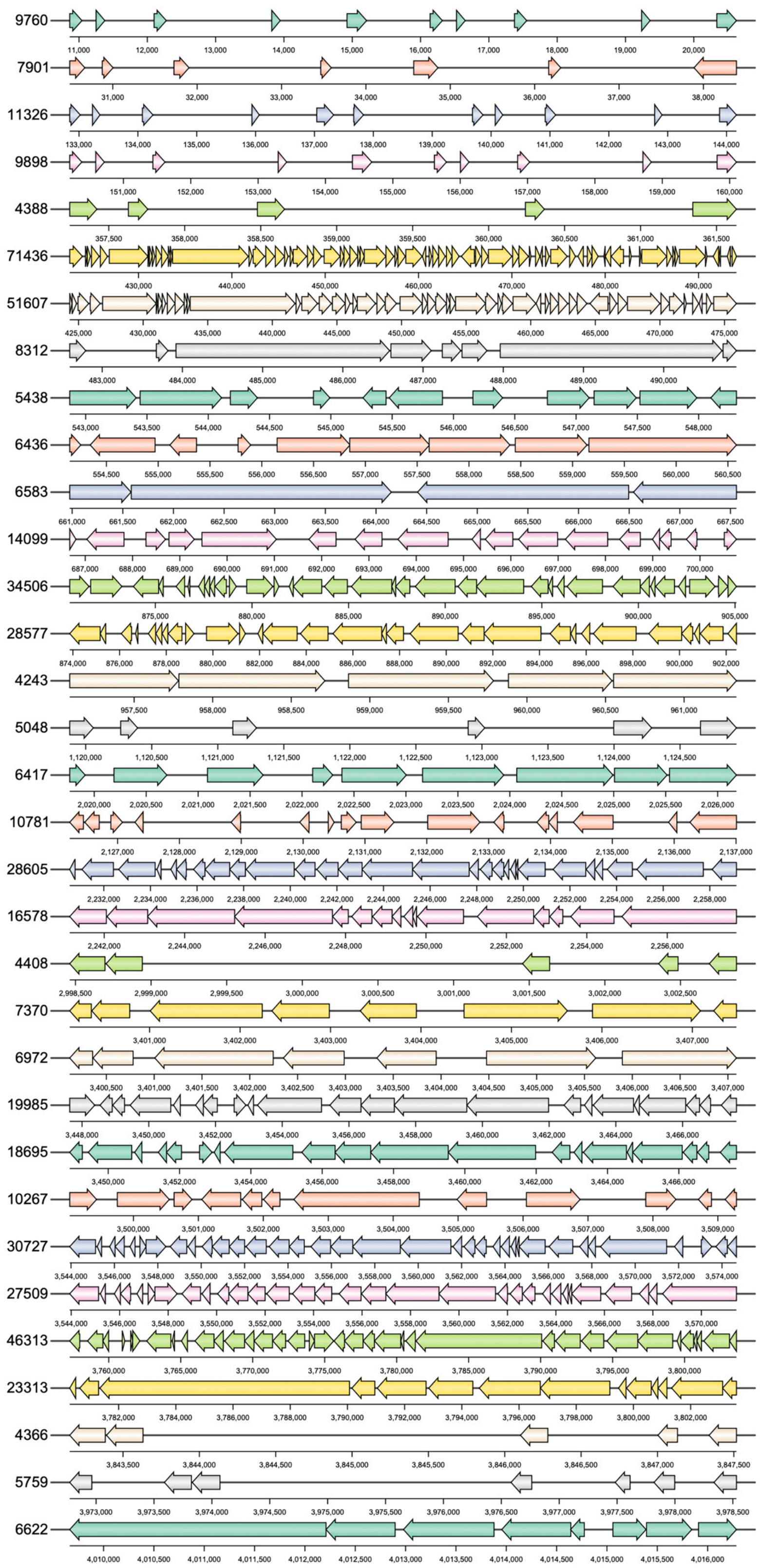

Figure S1: The statistical map of gene distribution in gene islands. The number before each genetic island represents the starting position of that genetic island on the chromosome. The direction of the arrow indicates the gene ORF on the sense or antisense strand. The horizontal scale indicates the proportional length of 1 kb of nucleotides on the chromosome. Different colors represent different genetic islands.

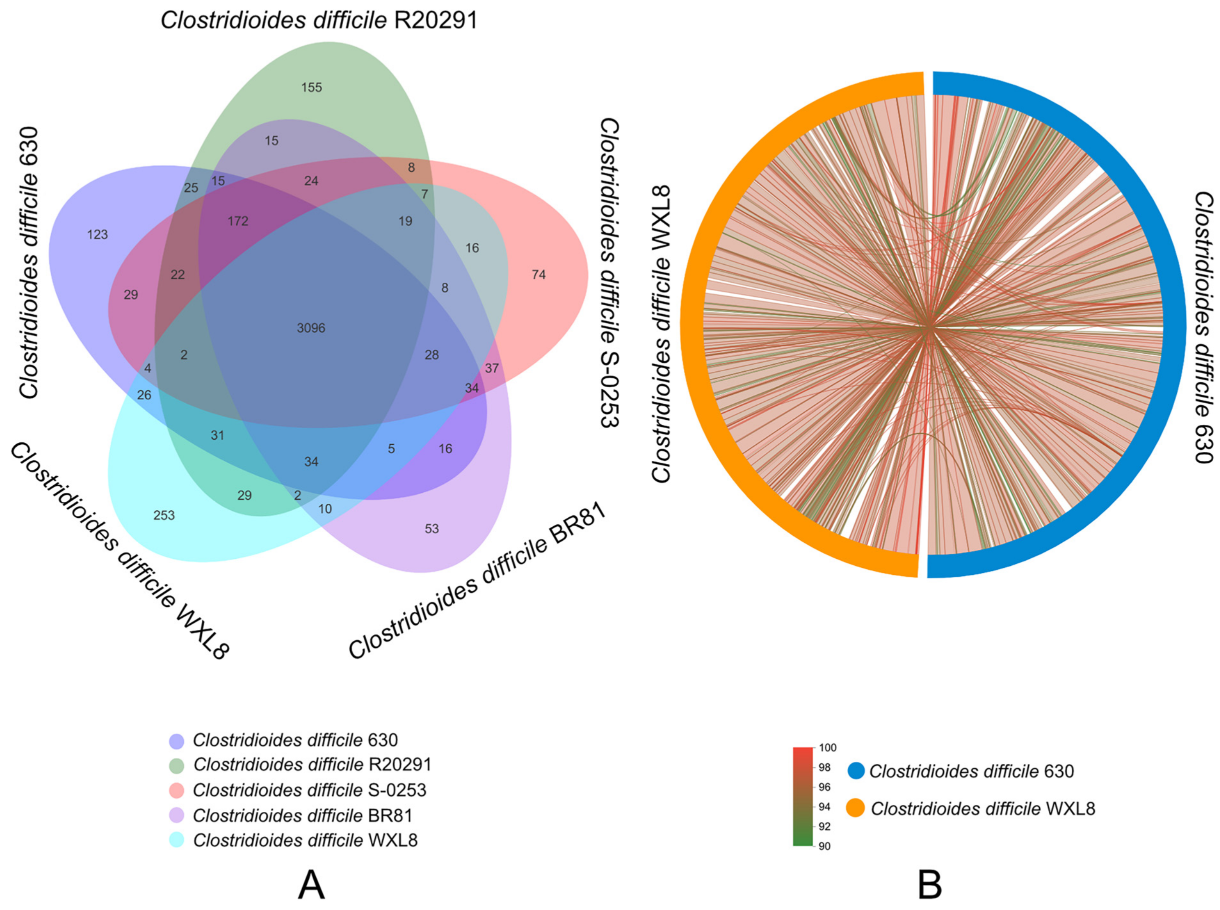

Figure S2: Comparative analysis of genomic features among various *Clostridioides difficile* strains. (a) The Venn diagram illustrates the overlap and distinctiveness in genomic characteristics of five *C. difficile* strains: 630, R20291, S-0253, BR81, and WXL8. Each circle corresponds to one strain; overlapping areas denote shared genes, while non-overlapping sections highlight unique genes specific to each strain. Numerical annotations indicate the quantity of shared or exclusive genomic features between these strains. (b) The Genome Collinearity Chord Diagram elucidates the genetic relationship between the WXL8 and *C. difficile* 630 strains. This diagram employs color-coded chords to represent connections between the two genomes:blue signifies *C. difficile* 630, while orange denotes WXL8. A gradient from red to blue is used for the chord colors, with bands indicating homology percentages ranging from 100 to 90%, as well as alignment lengths. Specifically, red corresponds to a homology range of 95–100%, and blue indicates 90–95%.
